# Elevated Temperature and Drought Interact to Reduce Parasitoid Effectiveness in Suppressing Hosts

**DOI:** 10.1371/journal.pone.0058136

**Published:** 2013-03-05

**Authors:** Cecilia M. Romo, Jason M. Tylianakis

**Affiliations:** School of Biological Sciences, University of Canterbury, Christchurch, New Zealand; University of California, Berkeley, United States of America

## Abstract

Climate change affects the abundance, distribution and activity of natural enemies that are important for suppressing herbivore crop pests. Moreover, higher mean temperatures and increased frequency of climatic extremes are expected to induce different responses across trophic levels, potentially disrupting predator-prey interactions. Using field observations, we examined the response of an aphid host-parasitoid system to variation in temperature. Temperature was positively associated with attack rates by parasitoids, but also with a non-significant trend towards increased attack rates by higher-level hyperparasitoids. Elevated hyperparasitism could partly offset any benefit of climate warming to parasitoids, and would suggest that higher trophic levels may hamper predictions of predator-prey interactions. Additionally, the mechanisms affecting host-parasitoid dynamics were examined using controlled laboratory experiments that simulated both temperature increase and drought. Parasitoid fitness and longevity responded differently when exposed to each climatic variable in isolation, compared to the interaction of both variables at once. Although temperature increase or drought tended to positively affect the ability of parasitoids to control aphid populations, these effects were significantly reversed when the drivers were expressed in concert. Additionally, separate warming and drought treatments reduced parasitoid longevity, and although temperature increased parasitoid emergence success and drought increased offspring production, combined temperature and drought produced the lowest parasitoid emergence. The non-additive effects of different climate drivers, combined with differing responses across trophic levels, suggest that predicting future pest outbreaks will be more challenging than previously imagined.

## Introduction

Climate change is expected to affect the presence, physiology, abundance, and distribution of plants, insect herbivores and their natural enemies [1]–[4]. These changes can be underpinned by effects of climate on specific life-history parameters such as development times and voltinism, and can affect population densities. Research on herbivores suggests that their performance, survival, and abundance can improve under conditions of simulated climate warming [5], as increases in temperature lead to faster development rates and increased voltinism [6]. In addition, consumption of plant foliage altered at elevated temperatures [1] could lead to changes in herbivore quality (e.g. chemical defenses or body size) or timing, indirectly affecting the fitness, abundance and activity of their natural enemies [7], [8].

In contrast to the effects of temperature alone, drought-induced changes to plant physiology (i.e. changes in plant secondary metabolites and nitrogen availability) [9]–[11] could alter the behavior and life-cycle parameters of insect herbivores [12]. Despite extensive work, there are still no clear predictions about the outcome of interactions between herbivores and drought-stressed plants [9], which can have either beneficial or detrimental effects on herbivore survival, development and population growth [6], [10] resulting from either enhanced diet or difficulty for herbivores in locating phloem, for example. Moreover, drought stress is likely to have variable effects on plants and herbivores due to the different ways in which plant species respond to drought, as well as differences among herbivores in their sensitivity to plant quality [13]. This uncertainty is further compounded by the potential for effects of drought on herbivores to depend on temperature or vice versa [14].

In addition to these highly-variable effects on herbivores, climate change is likely to disrupt higher-trophic-level relationships between organisms [14]–[16], as different members of a community can show differing responses [14], [17]. For example, increases in temperature alter insect phenologies, which can lead to a temporal and spatial mismatch between plants and herbivores or between herbivores and predators [18], [19]. Furthermore, predators are often more susceptible than other trophic levels to local and regional extinction following environmental changes [15]. Therefore, multi-trophic interactions will also be affected by climatic factors such as temperature and drought [10], [14], [20], which can drive different responses across species and trophic levels [17], [21], [22], potentially altering food webs and the stability of ecosystems [23], [24].

Although generalist predators may be less affected by altered phenology of a single prey species, specialist predators such as parasitoids often exhibit population dynamic responses that are strongly coupled with those of their host [25]. Parasitoids also play an important role in terrestrial ecosystems by influencing host abundance and dynamics, and perform a critical role as biological control agents of important pests in agroecosystems [26]. Although only a handful of empirical studies have compared the relative responses of interacting hosts and parasitoids to climate change [17], [27]–[29], theoretical models have shown that host-parasitoid relationships can be altered by environmental change. Temperature increase, for example, may differentially affect developmental rates of hosts and parasitoids [30]. Furthermore, any climate-related changes in the development rate of the host may affect their window of vulnerability to parasitism, thereby unravelling an intricate evolutionary history [8], [27], and altered host quality and population growth may affect host-selection by parasitoids [23]. Despite the ability of some parasitoids to acclimate to non-lethal high temperatures within one generation [8], prolonged heat stress can result in high mortality rates and reduced reproductive output [31]. Whether this heat stress makes them more vulnerable to dessication under drought conditions remains unknown. These examples highlight the potential vulnerability of parasitoids in the face of climate change, and emphasize the importance of understanding the mechanisms through which climate affects populations and parasitoid interactions with their hosts.

The effect of climate change on parasitoid-host interactions becomes more complex with the inclusion of higher-level predators (such as hyperparasitoids that attack primary parasitoids), which are also vulnerable to abiotic extremes, making them prone to local extinctions [32]. Research has shown that efficient herbivore control can be altered by the presence of hyperparasitoids [33], [34], though under field conditions this disruption may be ameliorated by disturbance [34]. However, there is little insight into how changes in climate will affect hyperparasitoids, which have important ramifications for top-down control of herbivores through trophic cascades. If variable responses among herbivores affect their interactions with primary parasitoids, extrinsic uncertainty in resource availability might be amplified up trophic chains, with sensitivity to climate differing across trophic levels [15].

Here we explore how biological control by parasitoids will respond in the face of an uncertain environmental future. Specifically, we used a model aphid-parasitoid-hyperparasitoid system to address the following questions:

1. How do the effects of climate at different trophic levels combine to affect the overall rates of parasitism?
2. How do different components of climate change (warming vs. drought) affect host-parasitoid interactions?
3. More importantly, how do responses of hosts and parasitoids to this combination of environmental drivers differ from their response to each climate component in isolation?

To answer these questions, we used a large-scale field study to address how temperature affects primary parasitism rates, hyperparasitism rates, and herbivore abundance, thereby testing whether climate effects are consistent across trophic levels. We then build upon these results using a series of laboratory experiments to tease apart the mechanisms that underpin population responses of parasitoids to altered climatic conditions, in the absence of higher-level predators (hyperparasitoids). These include the effects of drought as well as warming (temperature), and explore how these factors individually and combined affect parasitoid fecundity, longevity, emergence success and overall parasitoid fitness.

## Methods

### Ethics Statement

No specific permits were required for the described field studies. All field studies were performed with prior consent from each private landowner. Consent was provided by Bruce Carmichael, Greg Chamberlain, Kevin Clucas, James Guild, Trevor Hobson, Richard Maxwell, Peter Montgomery, Andrew Morris, Andrew Oram, and Warren Sheat.

### Study System

The dominant herbivore of kale (*Brassica oleracea* var. *acephala*) crops is the cabbage aphid (*Brevicoryne brassicae* (L.), Hemiptera), which is observed in the study region and supports a population of specialist parasitoid wasps (*Diaeretiella rapae* McIntosh, Hymenoptera: Aphidiinae). Their short development times and rapid asexual reproduction make aphids ideal organisms for testing population responses to global change [35]. However, the primary parasitoid species *D. rapae* is commonly seen among cabbage aphid colonies, and is an important source of aphid mortality. It is also the most ubiquitous parasitoid in our study region (C. Romo unpublished data) and the only parasitoid reared from *B. brassicae* in our samples, though a small number of *Aphidius ervi* parasitoids were reared from a nominal *Myzus persicae* population (data not presented). Importantly, *D. rapae* itself suffers from parasitism by other species of parasitic wasp called secondary parasitoids or hyperparasitoids, particularly *Alloxysta infuscata* (Kieffer) (Hymenoptera: Cynipidae), which was the only hyperparasitoid found parasitising *D. rapae* in our samples.

### Field Surveys of Aphid Abundance and Parasitism Rates Across a Natural Temperature Gradient

**Figure 1:**
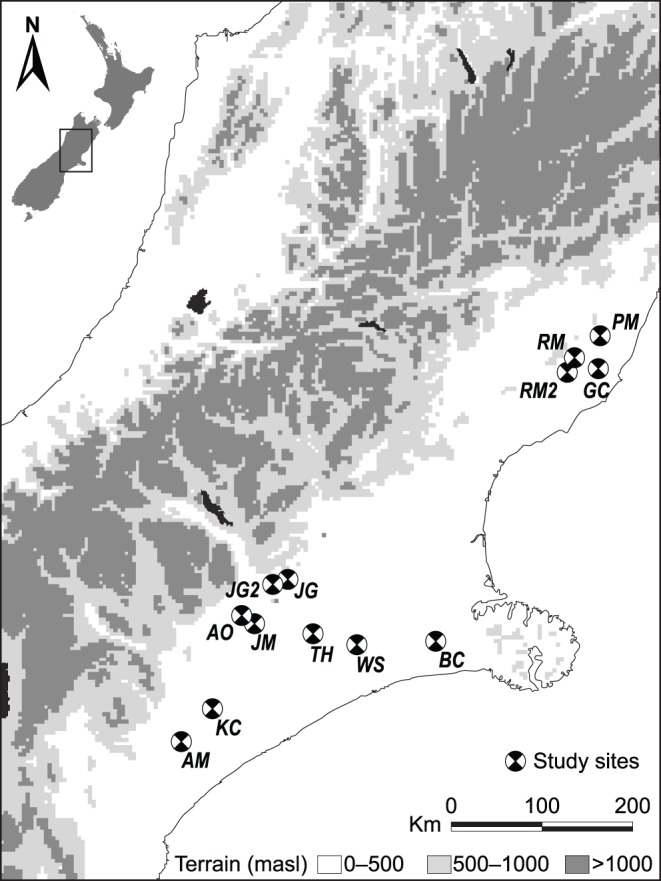
Field site locations. Markers indicate names and field site locations in Canterbury, South Island, New Zealand.

We used a large-scale field study to address how temperature affects parasitism and hyperparasitism rates and herbivore abundance. Our temperature gradient was generated predominantly by an elevation gradient from 5.4 to 517 meters a.s.l. Onset™ HOBO dataloggers were installed at each site to keep track of daily temperature fluctuations (highs/lows) and means for the duration of the experiment. During the summer of 2008–2009, we selected 13 sites (see Figure 1 and Table 1) throughout the Canterbury region, South Island, New Zealand, where kale *Brassica oleracea* var. *acephala* winter forage crops are widely grown. All sites were visited during the height of the growing season from March 20, 2009 to May 5, 2009 and all crops were conventional monoculture cropping systems, sown in late-November to early-December of the previous year. Daytime temperatures during the sampling period ranged from warm summer temperatures averaging 19°C in March, to cooler early autumn temperatures averaging 14°C in May. There was no significant latitudinal variation between sites (the northernmost site was approximately 217 km from the southernmost site), so there were negligible differences in day length across sites (maximum of 3.4 minutes by the end of sampling). Farming dominates the productive land in Canterbury, and the areas surrounding all sites were dominated by arable and pastoral land. Paddocks were between 6 and 10 hectares, and all sites were dryland farms (non-irrigated) that used no pesticides.

**Table 1:** Coordinates for all field sites located on the South Island, New Zealand, including average site temperature (±SE) during the sampling period with rainfall and altitude data.

Site name	Latitude (ddm)	Longitude (ddm)	Location	Avg. weekly temp (°C) (±SE)	Rainfall (mm)	Altitude
BC	S43°42.826′	E172°26.635′	Lincoln	13.27 (±0.51)	89.3	5.4
PM	S42°42.964′	E173°16.772′	Parnassus	12.03 (±0.51)	96.3	32
WS	S43°40.818′	E172°11.602′	Dunsandel	12.30 (±0.24)	95.8	70
GC	S42°50.1833′	E173°16.1332′	Cheviot	12.74 (±0.92)	64.7	105
RM1	S42°49.253′	E173°11.868′	Cheviot	12.80 (±0.88)	64.7	132
AM	S44°00.088′	E171°20.271′	Hinds	13.97 (±1.43)	87.0	172
KC	S43°52.770′	E171°32.077′	Ashburton	11.88 (±0.56)	125.2	173
TH	S43°39.501′	E171°55.588′	Rakaia	12.56 (±0.47)	89.4	188
RM2	S42°50.022′	E173°10.051′	Cheviot	13.18 (±0.59)	64.7	340
JM	S43° 36.814′	E171° 36.754′	Methven	11.03 (±0.39)	118.2	350
AO	S43°36.419′	E171°36.380′	Methven	10.58 (±0.40)	118.2	360
JG2	S43° 24.779′	E171° 47.456′	Windwhistle	10.78 (±0.70)	100.0	500
JG1	S43° 25.245′	E171° 45.974′	Windwhistle	10.60 (±0.60)	100.0	517

Over the course of the growing season, each site was sampled every 7–10 days for a total of 5 samples. We sampled sites using intensive area searches, which were conducted within a randomly-selected one square meter plot located at each site. A visual search for herbivorous insects was performed, and herbivores were taken back to the lab and reared for natural enemies (parasitoids). The major herbivore pest of brassicas in the region was the cabbage aphid (*Brevicoryne brassicae*: Hemiptera), for which we present data here.

#### Field data analysis

To test how aphid abundance and parasitism rates varied throughout the season at each site, and how this was related to temperature, we used generalized linear mixed effects models (GLMMs) with Poisson (for count data) or binomial (for proportion parasitism rates) error distributions. All analyses were conducted in the lme4 [36] and base package for R v.2.10 [37]. The testing of overdispersion in generalized mixed effects models is still the subject of some debate [38], however, it can be approximated using the ratio of the sum of squared Pearson residuals to the residual degrees of freedom. We counted each variance or covariance parameter as one model degree of freedom, and used the appropriate Chi square distribution [38] to test a null hypothesis that the ratio of Pearson residuals to degrees of freedom was zero. In cases where significant overdispersion was detected, we fitted observation-level random effects in GLMMs [39], but also present results from the models without these. We included site as a random effect, to allow for non-independence of multiple samples per site, and sampling round (1–5) as a categorical fixed predictor. We also included mean daily temperature in the month prior to sampling as a continuous fixed predictor, and included a temperature by time (sampling round) interaction. Because of the delay between a parasitism event and the observation of a parasitized aphid (‘mummy’), we used the mean temperature in the month prior to sampling as a measure of the temperature under which the parasitism event would have taken place. To account for possible density-dependence of parasitism rates, we included aphid abundance (at each sampling round) as a covariate. The maximal model including all of these variables was then simplified down to the minimal adequate model by removing non-significant interactions then main effects until no further improvement in fit (measured using the Akaike Information Criterion, AIC) was observed. During this model simplification procedure, models were fitted using maximum likelihood, whereas the final model was fitted using restricted maximum likelihood (REML) [40].

### Laboratory Mesocosm Study

#### Parasitism rate experiment

To provide a mechanistic explanation for temperature-associated changes in host and parasitoid population parameters observed in the field, we conducted a laboratory experiment in custom-built controlled-temperature Perspex™ mesocosms (80×80×80 cm), using treatments of ambient, 19±1°C, vs. elevated 23±1°C temperature and a 16/8 hour day/night cycle. Overnight temperatures for all mesocosms were set at 15°C ±1°C. These treatments were based on average summer daytime temperatures of about 19°C observed in the study region (Canterbury, New Zealand). In the next century, this region could expect an increase in average temperatures of at least 4°C as global surface temperatures rise [41], [42]. In addition, drought is another aspect of climate change that is expected to increase in frequency [41], worldwide and in our study region [43]. Therefore, we included a separate drought treatment (control vs. drought) in a factorial design with temperature. Control plants were watered with 150 ml every 3 days, which was enough to keep the plant hydrated and to keep the soil moist. For the drought treatment, drought incidence began at the start of the experiment and plants were watered with 75 ml, 3 times per week, resulting in short periods of pulsed drought stress [10]. We elected to impose drought in short bursts to allow some turgor recovery and to prevent plants from dying, and also because sap-feeding herbivores are highly susceptible to rapid changes in turgidity [9]. Also, simulating intermittent drought conditions is consistent with the potential increases of evapotranspiration expected in the east coast of New Zealand in the future [43]. I selected this watering regime for the drought treatments after preliminary trials indicated that this regime was sufficient for the plants to remain water stressed, while still allowing their survival and growth. Leaf water potential was measured with a pressure chamber (Peltier-effect thermocouple psychrometer) [44]. This revealed that overall, all plants underwent moderate levels of drought-stress (confirmed by readings of leaf water potential during the experiment). In total four mesocosms were used, with one for each treatment. Covered plants (including parasitoids in longevity experiment below) were rotated among mesocosms every four days to prevent any chamber effects. Despite this, plants in the same cage were non-independent, so these results should be interpreted with caution. All plants used were 3–4 week old *Brassica oleracea* var. *acephela* grown in controlled conditions (day: 22±1°C, night: 15±1°C, with a 16/8 hour day/night cycle). All aphid and parasitoid cultures used in all experiments were reared under the same controlled conditions prior to being added to factorial temperature and drought treatments. A total of 20 plants were used simultaneously, with five in each temperature × drought treatment combination. Four of the plants in each treatment contained parasitoids, whereas one control plant in each treatment had aphids only. The aphid-only treatment was simply intended to provide a baseline for qualitative comparison of parasitoid impact on aphid abundance (rather than to test the well-studied effects of temperature on herbivores [1], [45]), and was not included in the statistical models. Each of the five individual plants within each mesocosm was enclosed within a Mylar tube with Biomesh™ windows and covered with fine mesh bags (to prevent escape of aphids or parasitoids). Twenty late-instar cabbage aphids were placed on each plant and covered with clip cages [46] for 3 days to allow sufficient time for aphid colonies to establish. When clip cages were removed, a recently-emerged virgin female *Diaeretiella rapae* parasitoid was added to each plant in the parasitoid treatments. Each female wasp was left on the plant until its death (∼1 week old) with wasps being capable of attacking aphids from time of emergence [47]. Aphid abundance on each plant was counted every four days and the number of aphids parasitized was monitored daily. Upon daily inspection, adult parasitoids (haploid male offspring of the original female) were collected and removed to quantify parasitoid abundance and emergence success of the offspring. Parasitism rates were quantified by counting the number of parasitized aphid mummies across treatments. The trial ran for 26 days, once all offspring emerged (including 3 days after the last emergence for all treatments to account for any late emergence).

#### Parasitoid longevity experiment

Changes to parasitism rates under climate treatments could potentially be due to altered longevity of parasitoids, or altered parasitism rates per unit time (with time or longevity remaining constant). To separate these two mechanisms, we tested the effects of drought and temperature on parasitoid longevity. As longevity of parasitoids in the mesocosm experiment may have been affected by feeding on aphid honeydew [48], [49] or the number of oviposition events [50], we conducted a separate experiment from the parasitism experiment utilizing mummies from the same cohort to test for direct effects of temperature and drought on parasitoid longevity. *Diaeretiella rapae* mummies reared under control conditions were observed every hour during the day or every 4 hours at night (12 am–8 am) to check for emergence of adults. We individually reared hundreds of mummies, and at the point when enough mummies emerged within a 2-hour window (with any emerged wasps older than 2 hours being discarded), we were able to start the experiment. Upon emergence, individual adults were randomly placed in one of four treatments (elevated temperature, drought, elevated temperature+drought, control), ensuring that males and females were equally distributed in all treatments. Individual wasps were placed in cups with either wet or dry cotton rolls (control vs. drought), and placed into one of the four mesocosms. Wet cotton rolls were checked daily and kept wet. Wasps were checked every 6 hours until all adults died. Time from emergence until death was measured in hours and denoted as wasp longevity.

#### Laboratory data analysis

To test how aphid abundance varied through time in relation to temperature and drought, we used generalised linear mixed effects models (GLMMs) with Poisson errors. Parasitism rates and the proportion of parasitoids successfully emerging from mummies were analysed using generalised linear models (GLMs) with binomial error distributions. Temperature (elevated vs. ambient), drought (control vs. drought), and time were included as fixed predictors (with all possible interaction terms), and plant was included as a random effect in models of aphid population growth (to allow for non-independence of each time measurement from a given plant). The number of offspring per parasitoid was analyzed using a Poisson GLM with the total number of parasitoid mummies as the response variable, and temperature, drought and their interaction as predictors. Parasitoid longevity was analyzed using a linear model with time in hours as the response and drought, temperature, and sex (to allow for the possibility that males and females may differ in their survival time), plus their interactions, as predictors. We fitted observation-level random effects where significant overdispersion was detected [39]. Step-wise simplifications of each model (using AIC to compare fit of competing models at each step) were undertaken to find the best fit. Sex (and its interactions with drought and temperature) was eliminated from the model, resulting in a two-way ANOVA using temperature and drought as predictors. In all generalized models, the canonical link functions (logit for binomial and log for Poisson) were used.

## Results

### Field Results

#### Temperature correlations

**Figure 2:**
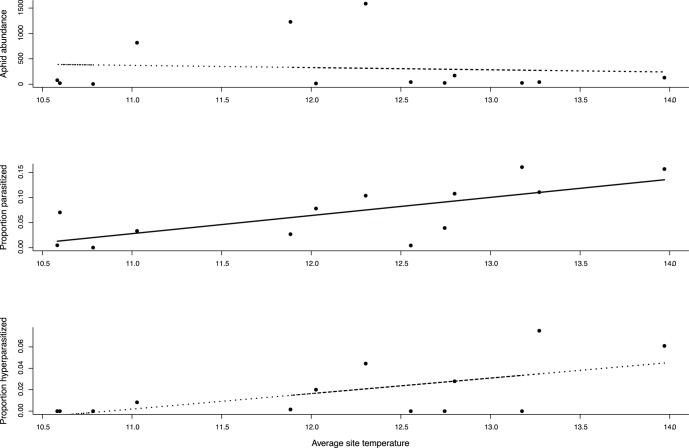
Temperature effects on each trophic level. Relationship between mean daily temperatures and (**A**) aphid abundance (*Z* = 0.18, *P* = 0.857), (**B**) proportion of aphids parasitised (*Z* = 5.91, *P*<0.001), and (**C**) proportion of aphids hyperparasitised (*Z* = 1.71, *P* = 0.087). Dashed lines indicate non-significant effects. Note: data are site averages, whereas individual measurement dates were analysed, grouped by sites, in the mixed effects models.

Aphids (n = 20,869) collected from the field varied in their abundance and parasitism rates in response to temperature through time (Table 2). Aphid abundances increased throughout the season within sites. However, this within-site population growth was marginally (though non-significantly) lower with increasing temperature (temperature × time interaction), such that aphid abundances were lower at the end of the season. Despite this, there was no significant main effect of temperature on aphid abundance (Table 2, Figure 2A).

**Table 2:** Results for GLMMs for all field data on the relationships between temperature and aphid population growth and parasitism.

Variable	Temperature		Time		Temperature × Time	
	*Z*	*P*	*Z*	*P*	*Z*	*P*
**Aphid abundance**	0.18	0.857	6.98	**<0.001**	−1.82	0.069
**Parasitism (proportion)**	5.91	**<0.001**	3.36	**<0.001**	−2.66	**0.007**
**Hyperparasitism (proportion)**	1.71	0.087	1.82	0.069	−1.98	0.066

Significant *P*-values are given in **bold**.

In contrast to aphid population growth, aphid primary parasitism (n = 947) increased as temperature increased, as did hyperparasitism (n = 501), though in the latter case the effect was marginally non-significant (Table 2, Figure 2B,C). Both primary and hyperparasitism (marginally non-significant) rates increased as the season progressed (Table 2). However the increases in primary parasitism throughout the growing season were lower under higher temperatures (temperature × time interaction), such that the effect of temperature weakened throughout the growing season (Table 2).

### Laboratory Results

#### Temperature effects

**Figure 3:**
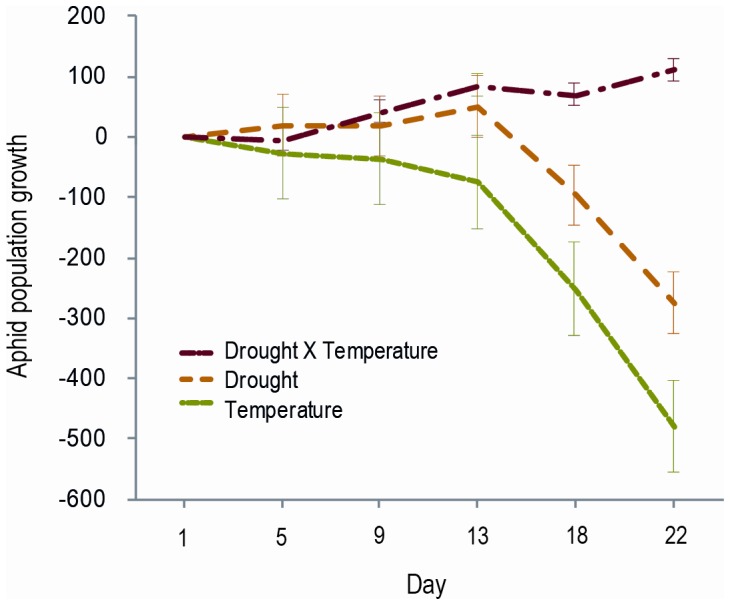
Interactive effects of temperature and drought on aphid population growth. Rate of aphid population growth in laboratory mesocosms under 3 treatments, calculated using aphid abundance in parasitoid (*Diaeretiella rapae*) treatments minus aphid abundances in the predator-free control to give the overall net predator effect (±SE). Temperature caused the greatest reduction in aphid population growth (*Z* = −4.87, *P*<0.001) followed by drought (*Z* = −5.92, *P*<0.001). In contrast, in the drought × temperature treatment (*Z* = 11.29, *P*<0.001) treatment, aphid population growth was positive.

**Figure 4:**
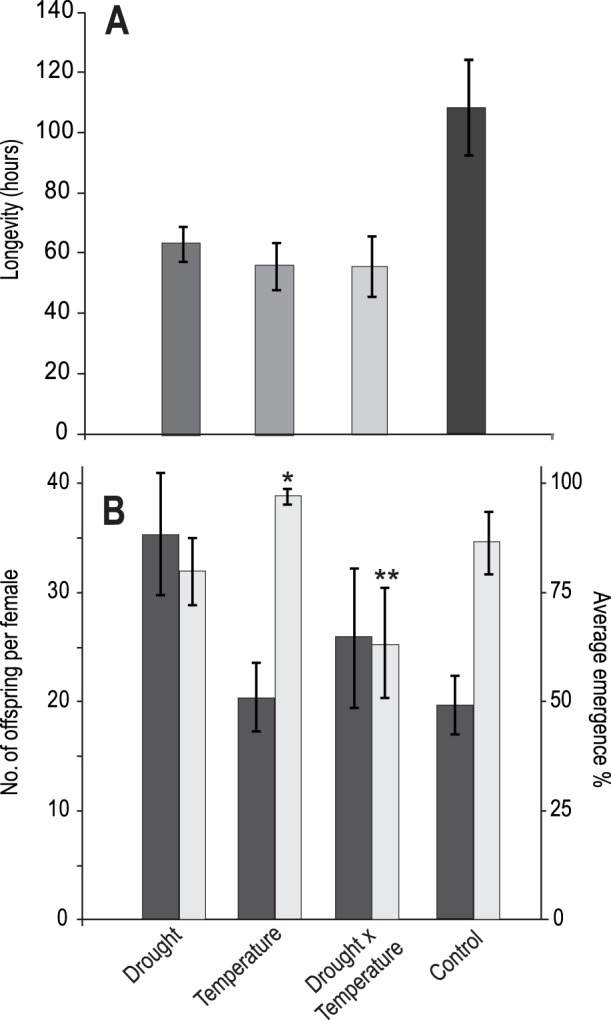
Effects of temperature and drought on parasitoid fitness. (**A**) Parasitoid longevity under drought, elevated temperature, drought and elevated temperature, and control treatments (±SE). (**B**) Number of offspring per female (dark bars) and percentage of successful adult emergence (light bars) (*temperature: *Z* = 2.29, *P* = 0.02, **drought × temperature: *Z* = −3.26, *P* = 0.001).

The parasitoid *D. rapae* was more effective (higher parasitism rates) under warmer temperatures, producing a negative effect on aphid population growth (Table 3; Figure 3), and thereby reducing aphids to below 30% of the abundance in the parasitoid-free control by the end of the experiment. However, the aphid population growth model appeared to be overdispersed, and the positive effect of temperature on aphid suppression by parasitoids became non significant when observation-level random effects were fitted to deal with overdispersion (Table 3). Thus, although parasitism rates were higher, the trend towards reduced aphid population growth should be interpreted with caution. Despite these apparent positive effects on parasitoids, adult parasitoid longevity was significantly reduced by temperature (Figure 4A), with adults in the warming treatment surviving for 55.8±7.7 hours, compared with 108.3±15.9 hours in the control treatment, an average net reduction of approximately 2.2 days or 51%. Elevated temperature did not significantly affect the number of parasitoid offspring (mummies) produced (Table 3), but it favored a significantly (*Z = *2.28, *P = *0.022) higher percentage of larval parasitoids successfully emerging (96% success compared with 85% in the control; Figure 4B).

**Table 3:** Results for all laboratory experiments on the effects of elevated temperature, drought, and their interaction, on parasitoid life-history parameters and aphid population response to parasitism using GLMMs, GLMs, and two-way ANOVAs.

Variable	Temperature		Drought		Temperature × Drought	
	*Z*	*P*	*Z*	*P*	*Z*	*P*
**Parasitism**	3.22	**<0.001**	3.64	**<0.001**	1.134	0.26
**Aphid population growth**	−4.87	**<0.001**	−5.92	**<0.001**	11.29	**<0.001**
fitted using OLR*	−0.04	0.362	−0.02	0.562	0.131	**0.017**
**No. of offspring**	0.079	0.937	4.12	**<0.001**	−1.55	0.12
**Emergence success**	2.28	**0.022**	−1.33	0.18	−3.26	**0.001**
	***t***	***P***	***t***	***P***	***t***	***P***
**Longevity**	−3.60	**0.002**	−3.25	**0.004**	2.20	0.043

Significant *P*-values are given in **bold**. *Using observation-level vector (OLV) as random effect to account for overdispersion.

#### Drought effects

*Diaeretiella rapae* was more effective at suppressing its host under drought conditions (negative effect on aphid population growth), and demonstrated significantly higher attack rates, though the trend towards reduced aphid population growth became non-significant when observation-level random effects were fitted (Table 3). Drought significantly reduced wasp longevity to 63.16±5.8 hours, compared to the control 108.3±15.9 hours, which is an average reduction in adult life by 1.9 days (Figure 4A). Despite this, there was a significant increase in the total number of offspring produced by each female *D. rapae* when exposed to drought conditions, with female wasps in the drought treatment laying on average 35±8 eggs, compared with only 19±4 in the control (Table 3). Despite this increased offspring production, there was no significant change in the proportion of those offspring that failed to emerge when exposed to drought conditions (Table 3; Figure 4B).

#### Temperature × drought interaction

Most importantly, the negative effects of elevated temperature and drought on aphid population growth were sub-additive, with a positive temperature × drought interaction term for aphid population growth outweighing the negative effects of each driver on growth (i.e. aphid populations grew faster than expected based on the separate effects of drought and temperature) in the presence of parasitoids (Figure 3; Table 3). Similarly, temperature and drought each significantly reduced parasitoid longevity, but their combined effect was sub-additive, such that when parasitoids were subjected to both temperature and drought, longevity was reduced to 55.6±10.2 hours (Figure 4A), which did not differ significantly from the effects of temperature alone. Male and female wasps did not differ significantly in their longevity, therefore sex was removed from this model.

Although temperature increased the emergence success of parasitoid offspring, and drought had a slight (non-significant) negative effect on emergence success, the negative temperature × drought interaction (*Z* = −3.26, *P = *0.001) saw significantly fewer parasitoids emerging when drought and temperature were combined than in any other treatment (Figure 4B).

## Discussion

Both our field and lab trials suggest that temperature changes can generate a disjunct between population growth rates of parasitoids and their hosts, which could alter many aspects of natural pest control in agricultural systems, analogously to altered consumer dynamics and effects on resource species seen in other systems (e.g. fish predators of zooplankton) [51]. Temperature is known to induce many changes to hosts and parasitoids that influence the population dynamics between them. As an illustration, elevated temperature significantly reduced adult parasitoid longevity by about 51% in our laboratory experiment. This could be attributed to an increase in metabolic rate [52], which is likely to mediate many of the biotic impacts of climate change globally [53]. Alternatively, higher temperatures or increased metabolic rates could simply influence the way parasitoids invest their energy. For example, parasitoids that experience thermal stress may opt to invest their effort into egg production to produce higher-quality offspring. A potential physiological mechanism for this is that, during the larval stage, a limited lipid reserve is available to a parasitoid that can be used for either egg production or adult survival (as most parasitoid species are incapable of *de novo* lipogenesis) [54]. Since high temperatures have been found to limit the amount of lipid accumulation, this temperature-induced restriction would lead to either an increased investment in offspring or in adult survival [54], [55]. For example, short periods (1 hour) of heat stress (36°C) resulted in high mortality of *Aphidius avenae*, and reduced reproductive output but greater longevity in surviving females [31], whereas in our study, *D. rapae* showed the opposite response to elevated temperature of reduced longevity but higher larval survival. The shift to either strategy in adults will be modulated by both resource availability and resource quality, which have the potential to change under varying environmental conditions.

The temperature effects and mechanisms we detected in the laboratory corresponded to our field data collected at larger spatial and temporal scales. Although previous work [56] suggests that temperature positively affects insect growth rates, our field survey found that aphid population growth tended (non-significantly) to be lowest at the warmest sites, congruent with long-term aphid population responses to temperature recorded by Adler et al. [57]. Also in accordance with laboratory results, parasitism rates in the field increased with temperature. The temperature correlation was strongest late in the season, which suggests that rapid parasitoid population growth, rather than initial attack rates, may have underpinned their ability to continuously suppress their host. Nevertheless, temperature is correlated with a number of other biotic and abiotic variables [58], so the field analyses must be interpreted as correlations, rather than necessarily as causal effects of temperature.

Given our findings for parasitoids, it is plausible that aphid abundance in the field was driven primarily by top-down parasitism pressure, which increased in the warmest sites and as the season progressed. This corresponds to periods when aphid population growth was at its lowest, and likely explains the trend towards a negative relationship between aphid population growth and temperature, which is known to be positive in the absence of top-down pressure [59]. Additionally, as emergence success of *D. rapae* increases at higher temperatures, this coupled with higher attack (i.e. reproductive) rates could lead to higher rates of parasitoid population growth during peak summer, and subsequent accumulation of parasitoid numbers late in the season in warmer sites. This accumulation through time was likely the reason for higher parasitism rates late in the season, despite lower autumn temperatures.

In addition to the potential disparity between the effects of climate on parasitoids vs. their hosts, the inclusion of higher-level predators can further affect the efficacy of parasitoids as control agents in the field. We found that temperature was marginally related with hyperparasitism, which suggests that climate change may also enhance the fourth trophic level, potentially releasing herbivores from attack via a trophic cascade. Although this field study only aimed to study the effects of temperature, the addition of water-stressed conditions could potentially create entirely different outcomes, as our laboratory experiments emphasized.

Our mesocosm experiments showed that when temperature or drought treatments were applied separately, parasitism rates increased, producing a trend towards stronger suppression of aphid population growth. These changes in attack rates might suggest that drought in the absence of temperature increases could contribute to increases in parasitoid populations, but the effects of drought on emergence success counteract this view. We found that the emergence success of *D. rapae* was significantly lower on water-stressed plants, congruent with previous work showing negative impacts of drought on parasitoid abundances [60].

The positive effects of temperature on parasitoids we observed here will obviously have an upper limit, beyond which survival will be threatened. Indeed, physiological stress of another aphidiine parasitoid (*Aphidius matricariae*) on wheat aphids has been shown to prevent adult emergence at high temperatures (31°C), even though parasitoid development rate increased linearly with increasing temperature between 11 and 26°C [61]. Therefore, the net effect of temperature on host suppression will depend on the difference between host and parasitoid temperature tolerance. Although previous work suggests that parasitoids have higher minimum temperature thresholds than their hosts [62], which would allow them to benefit more from warmer spring temperatures, this may not translate into higher tolerance thresholds for maximum temperatures. Nevertheless, a recent study of *A. matricariae* on green peach aphid demonstrated that heat stress (32–40°C heatwaves either biweekly or daily) reduced aphid population growth and increased the costs to aphids of behavioral responses to foraging parasitoids [63], whereas there was no evidence that temperature extremes and ﬂuctuations affected parasitoid foraging ability. This suggests that parasitoids may indeed incur a net benefit over their hosts under high temperatures in the absence of drought.

From a pest suppression perspective, increased attack rates under elevated temperature or drought are a positive outcome, but these benefits were reversed for host suppression when temperature increase and drought acted in concert. Likewise, the negative effect of simulated drought on emergence success of the parasitoid was exacerbated by high temperatures, adding support to the argument that predictions based on studies of single environmental change drivers may not hold in the presence of other drivers [14]. Treatments with both drivers experienced over 35% mortality of larval parasitoids, suggesting that despite producing greater absolute numbers of offspring, these offspring were potentially of inferior quality. Although high temperatures and drought alone had positive or slightly negative (respectively) effects on the total number of parasitoid offspring, the combined stress of both drivers produced high mortality of larval/pupal parasitoids, suggesting that either larval parasitoids were too stressed physiologically to successfully pupate, or that prey quality was reduced suffciently to inhibit pupation under the combined effects of the drivers.

Aphid quality is an important factor that will either limit or enhance parasitoid fitness. Although our experiment was not designed to measure aphid responses in the absence of parasitoids, previous work has shown positive aphid responses to drought [64]. Any benefits to aphids should have translated into higher-quality hosts for parasitoids to exploit, leading to enhanced parasitoid fecundity and emergence success. Although females oviposited more often under drought conditions, the emergence success of those offspring did not differ significantly from those under control conditions.

### Conclusions

We found that the herbivore and parasitoid had different responses to temperature and drought, with primarily negative top-down effects on aphids when exposed to either temperature or drought separately. However, insect responses to climatic changes in isolation can differ considerably from their responses to the interaction of multiple components of climate change. Indeed, our findings for parasitoid-host interactions under warming and drought contrasted with predictions based on the singular effects of individual drivers. These changes could affect the role of parasitoids in the suppression of pests in outbreaks. Studying these combined drivers offers a realistic view of what ecosystems and biological control programmes will face under a regime of climate change.
